# A Probabilistic Framework for Finite Strain Damage Response of Thick Curved Beams Including the Shear Effect

**DOI:** 10.3390/ma19143073

**Published:** 2026-07-16

**Authors:** Arian Mohammadkhani, Hamid Shahsavari, Mostafa Baghani

**Affiliations:** 1 School of Mechanical Engineering, College of Engineering, University of Tehran, Tehran 14399-57131, Iran; arian.m.khani@ut.ac.ir; 2 Department of Surgery, University of Oklahoma Health Sciences Center, Oklahoma City, OK 73104, USA

**Keywords:** curved beam, hyperelasticity, mullins effect, probabilistic analysis, nonlinear solid mechanic, finite element method

## Abstract

Curved beams are widely used in engineering applications due to their unique geometry and mechanical advantages. In this study, the mechanical response of hyperelastic curved beams under cyclic loading is investigated, considering the Mullins stress-softening effect and the probabilistic variability of material parameters. A Neo-Hookean hyperelastic model integrated with the Ogden–Roxburgh damage formulation is employed to capture the cyclic stress-softening behavior. The governing partial differential equations are derived in cylindrical coordinates under plane-stress conditions and solved numerically. Unlike classical pure bending assumptions, the present formulation captures the coupled radial, circumferential, and shear stress components arising from the finite thickness and curvature of the beam. The model is validated through comparison with two-dimensional finite element solutions. The influence of material uncertainty is further examined via a probabilistic parametric analysis, and statistical measures including mean, standard deviation, skewness, and kurtosis are evaluated. The results demonstrate that Mullins-induced stress softening is concentrated near the inner curvature and beam root, and that inherent material variability significantly affects the damage distribution. The proposed framework provides a fast and accurate method for predicting the mechanical behavior of hyperelastic curved beams under cyclic loading.

## 1. Introduction

Curved beams, due to their special geometry, have been widely used in various industries from past to present [1,2,3,4]. These applications range from structural reinforcements in aerospace systems to metal hooks and chains. Nowadays, manufacturing curved beams using polymers can significantly expand their applications. For instance, a chain made of polymer has much higher flexibility compared to its metallic counterpart, and in addition, has the ability to absorb vibrations due to its viscoelastic properties [5,6]. These features can play an important role in designing lighter, stronger structures with enhanced shock and vibration absorption capacity [7,8,9,10,11]. Unlike most analyses on polymeric beams, which typically assume pure bending [12,13,14,15], in this study, the mechanical response of hyperelastic curved beams under general bending conditions is investigated. Due to the finite thickness and curvature, the beams exhibit not only circumferential and radial normal stresses but also non-negligible shear stresses, which significantly influence their overall mechanical behavior.

Furthermore, the use of polymers in the construction of curved beams opens up new applications. Today, polymers are replacing metallic materials due to their advantages such as low cost, printability, high ductility, etc. New polymers are widely used in applications such as the manufacture of various sensors [16,17,18], aerospace [19], and medical stents [20,21]. Since polymer materials are composed of molecular chains [22,23], these chains undergo rearrangement and debonding during each loading cycle. Consequently, in successive loadings, polymers sustain damage and their stiffness degrades. For this reason, the analysis of polymer damage under cyclic loading is of particular importance [24,25]. The softening of polymers under repeated loading cycles is known as the Mullins effect [26], and it can be evaluated using various mathematical formulations [27,28,29,30,31]. However, all these formulations depend on material parameters. Since these parameters exhibit inherent variability even in nominally identical materials, they must be treated as statistical quantities. By incorporating statistical distributions, the structural responses transition from deterministic values to probabilistic measures, where each outcome is quantified by its likelihood. Consequently, this probabilistic framework offers a more realistic and high-fidelity representation of the actual material behavior [32,33].

Since curved beams, unlike straight beams, have more complex stress and strain distributions, studying their behavior under different loading conditions is of great importance. Accurate analysis of these behaviors can lead to more optimized designs, extended service life of structures, reduced maintenance costs, and improved overall performance of engineering systems [34]. Since the introduction of curved beam theory, numerous studies have been conducted on metallic beams with elastic behavior. For example, Rajasekaran and Padmanabhan (1989) presented more accurate equations for thin-shell curved beams using the principle of virtual work and large displacement theory [1]. Likewise, Pi and Trahair (1997) investigated the nonlinear behavior of in-plane curved I-beams, and by developing a finite element model, they demonstrated that at large curvature angles, torsion and bending simultaneously and nonlinearly influence the beam’s response [35]. Litewka and Rakowski (1997) developed an efficient two-node finite element for curved beams which, by eliminating shear and membrane locking, provided high accuracy and good convergence even with coarse meshes [36]. Fazlali et al. presented an analytical solution based on hyperbolic strain distribution to study the elastoplastic pure bending response of curved beams with linear kinematic hardening under loading and unloading. They compared and validated the obtained results against detailed numerical analyses [37]. In the field of curved beam vibrations, Chidambaram and Lisa (1993) analyzed the oscillations of planar curved beams and arches, investigating the effects of initial loads, nonlinear behavior, and finite element modeling [4]. Xiang and Chiang (2004) proposed a simplified method for deriving the stiffness and mass matrices of circular Timoshenko curved beam elements, incorporating rotary inertia and shear deformation effects in free vibration modeling, and validated the accuracy of their results against analytical and numerical solutions [38].

Although most of these studies focused on metallic curved beams, there have also been investigations into non-metallic ones. For instance, Lin and Hsieh (2007) [39] presented closed-form analytical solutions for two-dimensional laminated curved beams with variable curvature. By decomposing components using harmonic functions, they analyzed their mechanical behavior and demonstrated that the results for isotropic beams were consistent with existing solutions. They further examined the influence of parameters such as thickness ratio, layer stacking sequence, and geometric forms (e.g., elliptical, parabolic, catenary, and helical) on structural responses [39]. More recent research with different perspectives has also emerged. Barretta, Marotti de Sciarra, and Vaccaro (2019) [40] modeled the static behavior of curved elastic beams at the nanoscale using stress-driven nonlocal mechanics. They showed that size effects lead to stiffening in nano-structures, making this approach suitable for the design of nano-sensors and actuators [40]. Curved viscoelastic beams have also been examined in several studies. Pivan and Cortínez (2008) modeled the bending–torsion behavior of straight and curved thin-shell composite beams using the correspondence principle and linear viscoelastic analysis, and validated the finite element results in the Laplace domain against experimental data [41]. Erkmen and Bradford (2011) [42] developed a numerical model to study the time-dependent creep and shrinkage behavior of in-plane steel–concrete composite curved beams. By employing the Wiechert viscoelastic model, they incorporated the effects of concrete aging and partial interaction of connections on long-term bending behavior, and validated the results against detailed finite element models and available experimental data [42].

However, to date, no study has comprehensively examined the behavior of hyperelastic curved beams under cyclic boundary conditions. The primary objective of the present work is to address this gap and provide a statistical analysis of their mechanical response under such loading conditions.

## 2. Theoretical Method

For beams with an initial curvature, the bending theory must also include effects of curvature [43,44,45,46]. Two important differences with respect to straight beam bending result. First, the stress distribution across the beam thickness becomes nonlinear along the radial direction due to curvature. Consequently, the neutral axis will not coincide with the centroidal axis of the cross section when the beam is subjected to pure bending. Second, a curved beam carries radial stress because of the internal bending moment. These radial stresses have important design implications for thin-walled cross sections and for materials (such as unidirectional composites) with relatively low tensile strength in the radial direction. In this section, the governing equations of a thick curved beam are investigated. Due to the in-plane (radial) thickness and the curvature of this beam, the shear stress exhibits a significant magnitude, in addition to the hoop and radial stresses. Furthermore, it should be noted that owing to the small thickness of the beam in the out-of-plane direction (perpendicular to the plane of bending), the equations are formulated based on the plane stress assumption.

Furthermore, due to the cyclic displacement applied to the hyperelastic curved beam, the Mullins effect, represented by a damage function, has been incorporated into the material model’s energy function. This is due to the stiffness changes observed across different cycles. Overall, this section presents a comprehensive formulation for an incompressible hyperelastic curved beam with a significant radial thickness, systematically incorporating the Mullins stress-softening effect under cyclic loading.

### 2.1. Kinematics of Curved Beams

**Figure 1:**
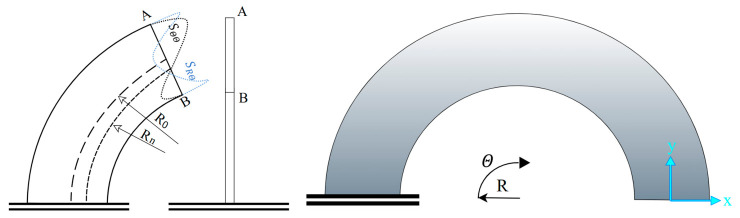
Schematic diagram of the geometry of a thick, curved beam with reference and current coordinates, where A and B represent the cross-sections of the beam; the thick dimension is in the R−Θ plane and the thin dimension is in the direction perpendicular to it.

For a curved beam under plane stress conditions, the reference configuration is described in cylindrical coordinates (R,Θ,Z) due to the initial curvature of the beam (Figure 1). However, in the current (deformed) configuration, the beam’s deformation (particularly the vertical displacement at its free end) is more conveniently described in Cartesian coordinates (x,y,z). Accordingly, the motion of a material point is defined through a mapping from the reference configuration to the current configuration. Let (R,Θ) denote the initial coordinates of a point in the reference state. Upon deformation, the point moves to a new position characterized by the current radial coordinate r and the deformed angular coordinate θ. Assuming plane stress conditions, the position vector of a material point in the current configuration is expressed as:

(1) x(R,Θ)=r(R,Θ)cosθ(R,Θ)

(2) y(R,Θ)=r(R,Θ)sinθ(R,Θ)

In the reference configuration, $R_{0}$ denotes the distance between the center of the cross section and the center of curvature and $R_{n}$ denotes the neutral axis. Under plane stress conditions, the displacement components in cylindrical coordinates are expressed as

(3) $$u_{r}=r-R,\;u_{\theta }=R\left(\theta -\Theta \right)$$

where $u_{r}$ and $u_{\theta }$ denote the radial and tangential displacement components, respectively. The corresponding displacement components in the Cartesian coordinate system are related to their cylindrical counterparts through

(4) $$u_{x}=u_{r}cos\Theta -u_{\theta }sin\Theta$$

(5) $$u_{y}=u_{r}sin\Theta +u_{\theta }cos\Theta$$

For hyperelastic materials, the deformation gradient tensor is required to describe finite strain kinematics [47]. It is defined as

(6) $$F=I+\nabla u=\frac{\partial x}{\partial X}$$

where X=(R,Θ,Z) and x=(x,y,z) denote the position vectors in the reference and current configurations, respectively. Since the reference configuration is described in cylindrical coordinates, while the current configuration is expressed in Cartesian coordinates, the deformation gradient admits a mixed cylindrical–Cartesian representation. Under the assumptions of plane stress conditions and no out-of-plane shear deformation, the deformation gradient tensor takes the form

(7) $$F=\left[\begin{array}{ccc} x_{,R} & \frac{1}{R}x_{,\Theta } & 0\\ y_{,R} & \frac{1}{R}y_{,\Theta } & 0\\ 0 & 0 & z_{,Z} \end{array}\right]=\left[\begin{array}{ccc} r_{,R}cos\theta -r\theta_{,R}sin\theta & {\frac{1}{R}(r}_{,\Theta }cos\theta -r\theta_{,\Theta }sin\theta ) & 0\\ r_{,R}sin\theta +r\theta_{,R}cos\theta & {\frac{1}{R}(r}_{,\Theta }sin\theta +r\theta_{,\Theta }cos\theta ) & 0\\ 0 & 0 & z_{,Z} \end{array}\right]$$

For incompressible hyperelastic materials, the constraint det F=1 is imposed. Enforcing this condition yields the stretch in the out-of-plane direction as

(8) $$\lambda_{z}=z_{,Z}=\frac{R}{r\left(r_{,R}\theta_{,\Theta }-r_{,\Theta }\theta_{,R}\right)}$$

The strain energy density function for hyperelastic materials is expressed in terms of the deformation measures, which characterize local stretches and rotations. These measures are commonly represented by the right and left Cauchy–Green deformation tensors, or equivalently by the principal stretches. The right Cauchy–Green deformation tensor is defined as:

(9) $$C=F^{T}F=\left[\begin{array}{ccc} r_{,R}^{2}+r^{2}\theta_{,R}^{2} & \frac{1}{R}\left(r_{,R}r_{,\Theta }+r^{2}\theta_{,R}\theta_{,\Theta }\right) & 0\\ \frac{1}{R}\left(r_{,R}r_{,\Theta }+r^{2}\theta_{,R}\theta_{,\Theta }\right) & \frac{1}{R^{2}}\left(r_{,\Theta }^{2}+r^{2}\theta_{,\Theta }^{2}\right) & 0\\ 0 & 0 & \lambda_{z}^{2} \end{array}\right]$$

The inverse of the right Cauchy–Green deformation tensor ($C^{-1}$), can be obtained accordingly and is used in the subsequent constitutive formulation.

(10) $$C^{-1}=\left[\begin{array}{ccc} \frac{r_{,\Theta }^{2}+r^{2}\theta_{,\Theta }^{2}}{r^{2}(r_{,R}\theta_{,\Theta }-r_{,\Theta }\theta_{,R}{)}^{2}} & -\frac{R(r_{,R}r_{,\Theta }+r^{2}\theta_{,R}\theta_{,\Theta })}{r^{2}(r_{,R}\theta_{,\Theta }-r_{,\Theta }\theta_{,R}{)}^{2}} & 0\\ -\frac{R(r_{,R}r_{,\Theta }+r^{2}\theta_{,R}\theta_{,\Theta })}{r^{2}(r_{,R}\theta_{,\Theta }-r_{,\Theta }\theta_{,R}{)}^{2}} & \frac{R^{2}(r_{,R}^{2}+r^{2}\theta_{,R}^{2})}{r^{2}(r_{,R}\theta_{,\Theta }-r_{,\Theta }\theta_{,R}{)}^{2}} & 0\\ 0 & 0 & \frac{1}{\lambda_{z}^{2}} \end{array}\right]$$

### 2.2. Constitutive Equation

In this study, the Neo-Hookean strain energy function is considered [48,49], as shown in Equation (11), where $c_{1}$ is a material constant related to the shear modulus, and $I_{1}=\mathrm{tr}(C)$ is the first invariant of the right Cauchy–Green deformation tensor.

(11) $$\Psi =-p\left(j-1\right)+c_{1}\left(I_{1}-3\right)$$

The Second Piola–Kirchhoff stress tensor is derived from the strain energy function as Equation (12), where p is the indeterminate Lagrange multiplier.

(12) $$S_{0}=-pC^{-1}+2\frac{\partial \Psi }{\partial C}=-pC^{-1}+2c_{1}I$$

The individual components of the Second Piola–Kirchhoff stress tensor in the mixed cylindrical representation are therefore expressed as

(13) $${S_{0}}_{RR}=-p{C^{-1}}_{11}+2c_{1}$$

(14) $${S_{0}}_{\Theta \Theta }=-p{C^{-1}}_{22}+2c_{1}$$

(15) $${S_{0}}_{zz}=-p{C^{-1}}_{33}+2c_{1}$$

(16) $${S_{0}}_{R\Theta }=-p{C^{-1}}_{12}$$

Under plane-stress conditions, the out-of-plane Cauchy stress component must vanish in the current configuration, that is, $\sigma_{zz}=0$. The Cauchy stress tensor σ is related to the Second Piola–Kirchhoff stress tensor S through the standard push-forward relation $\sigma =\frac{1}{J}FSF^{T}$. Accordingly, the zz-component of the Cauchy stress can be written as $\sigma_{zz}=\frac{1}{J}e_{z}^{T}FSF^{T}e_{z}$. For the present deformation, assuming that the out-of-plane direction remains uncoupled from the in-plane deformation so that $F_{zR}=F_{z\Theta }=F_{Rz}=F_{\Theta z}=0$ and $F_{zz}=\lambda_{z}$, this relation simplifies to $\sigma_{zz}=\frac{\lambda_{z}^{2}}{J}S_{zz}$. Therefore, since J=1 and $\lambda_{z}\neq 0$, the plane-stress condition $\sigma_{zz}=0$ directly implies that $S_{zz}=0$. This result then allows the pressure-like Lagrange multiplier p to be determined:

(17) $${S_{0}}_{zz}=0\to p=\frac{2c_{1}}{{C^{-1}}_{33}}=2c_{1}{\lambda_{z}}^{2}$$

The components of σ in the Cartesian spatial system (x,y,z) can thus be directly obtained from the known deformation gradient and the computed Second Piola–Kirchhoff stresses. This formulation allows for direct interpretation of stress distribution in the deformed (current) configuration.

### 2.3. Mullins Effect

To incorporate stress-softening (Mullins) behavior into the Neo-Hookean model, the Ogden–Roxburgh damage formulation [26] is adopted. The modified strain energy density function is written as

(18) $$\Psi_{\left(I_{1},\eta \right)}=\eta \Psi_{\left(I_{1}\right)}+\Phi_{\left(\eta \right)}$$

where $\Psi_{\left(I_{1}\right)}$ is the conventional neo-Hookean energy function which is scaled by softening factor η. Also, the energy density $\Phi_{\left(\eta \right)}$ accounts for the softening energy spent during loading and unloading.

(19) $$\eta =1-\frac{1}{R}erf\left(\frac{\Psi_{\left(max\right)}-\Psi }{m+\beta \Psi_{\left(max\right)}}\right)$$

And the energy density associated with softening is

(20) $$\Phi_{\left(\eta \right)}=\frac{m+\beta \Psi_{\left(max\right)}}{R\sqrt{\pi }}\left[exp{\left(\frac{\Psi_{\left(max\right)}-\Psi }{m+\beta \Psi_{\left(max\right)}}\right)}^{2}-1\right]+(1-\eta )\Psi_{\left(max\right)}$$

where $\Psi_{\left(max\right)}$ is the maximum strain-energy density previously reached during the loading history. As the material is loaded beyond its historical maximum, η approaches 1 and the behavior becomes purely hyperelastic; during unloading, the argument of the error function increases, leading to η<1, which produces stress-softening. The parameters m, β, and r are material constants that control the degree of softening and recovery during cyclic loading.

Finally, the modified Second Piola–Kirchhoff stress incorporating the Mullins effect is then given by

(21) $$S=\eta S_{0}$$

where $S_{0}$ is the second Piola–Kirchhoff stress tensor of the Neo-Hookean material without damage.

### 2.4. Equilibrium Equations

To obtain the displacement field, it is necessary to apply the equilibrium equations. In the absence of body forces and acceleration, the equilibrium equations reduce to the following condition:

(22) Div S=0

In the reference cylindrical coordinates, the equilibrium equations yield the following governing equations:

(23) $$\frac{\partial S_{RR}}{\partial R}+\frac{1}{R}\frac{\partial S_{R\Theta }}{\partial \Theta }+\frac{S_{RR}-S_{\Theta \Theta }}{R}=0$$

(24) $$\frac{\partial S_{R\Theta }}{\partial R}+\frac{1}{R}\frac{\partial S_{\Theta \Theta }}{\partial \Theta }+\frac{2S_{R\Theta }}{R}=0$$

By substituting the Second Piola–Kirchhoff relation (including the Mullins effect, see Equation (21)) into Equations (23) and (24), two PDE coupling equations are obtained. By solving these equations, the displacement field was evaluated, and using the displacement field, the stress was determined. Since η is a scalar assumed spatially uniform within each load step (i.e., ∇η=0), the equilibrium operator satisfies $Div\;S=\eta \;Div\;S_{0}=0.$ Therefore, the governing PDEs are identical to those obtained for $S_{0}$, with η entering only as a multiplicative factor in the stress update.

(25) $$\frac{\partial }{\partial R}(\frac{R^{2}{(r}_{,\Theta }^{2}+r^{2}\theta_{,\Theta }^{2})}{r^{4}(r_{,R}\theta_{,\Theta }-r_{,\Theta }\theta_{,R}{)}^{4}})-\frac{1}{R}\frac{\partial }{\partial \Theta }(\frac{R^{3}\left(r_{,R}r_{,\Theta }+r^{2}\theta_{,R}\theta_{,\Theta }\right)}{r^{4}(r_{,R}\theta_{,\Theta }-r_{,\Theta }\theta_{,R}{)}^{4}})+R(\frac{r_{,\Theta }^{2}+r^{2}\theta_{,\Theta }^{2}-R^{2}\left(r_{,R}^{2}+r^{2}\theta_{,R}^{2}\right)}{r^{4}(r_{,R}\theta_{,\Theta }-r_{,\Theta }\theta_{,R}{)}^{4}})=0$$

(26) $$\frac{\partial }{\partial R}\left(\frac{R^{3}\left(r_{,R}r_{,\Theta }+r^{2}\theta_{,R}\theta_{,\Theta }\right)}{r^{4}(r_{,R}\theta_{,\Theta }-r_{,\Theta }\theta_{,R}{)}^{4}}\right)-\frac{1}{R}\frac{\partial }{\partial \Theta }\left(\frac{R^{4}\left(r_{,R}^{2}+r^{2}\theta_{,R}^{2}\right)}{r^{4}(r_{,R}\theta_{,\Theta }-r_{,\Theta }\theta_{,R}{)}^{4}}\right)+2\frac{R^{3}(r_{,R}r_{,\Theta }+r^{2}\theta_{,R}\theta_{,\Theta })}{r^{4}(r_{,R}\theta_{,\Theta }-r_{,\Theta }\theta_{,R}{)}^{4}}=0$$

These coupled PDEs are solved numerically to obtain the displacement field. Subsequently, stress components are evaluated using the known displacement field and the constitutive relations.

## 3. Results

**Figure 2:**
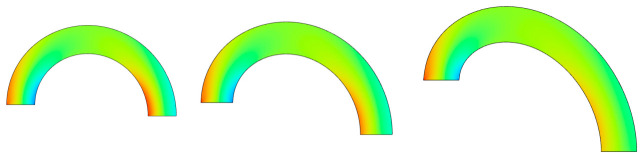
Deformed shape of the curved beam in 2D FEM with the Cauchy stress contour in the $\sigma_{yy}$ direction.

**Figure 3:**
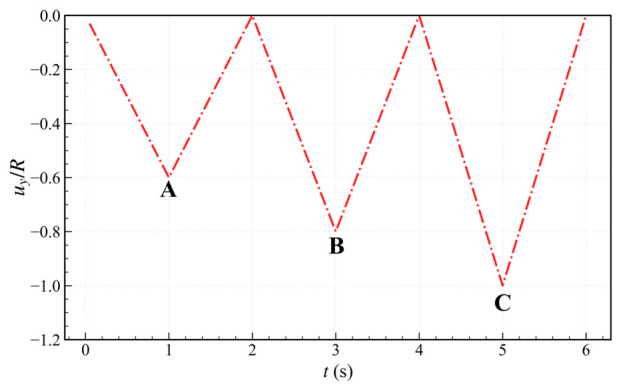
Cyclic vertical displacement normalized by the radius of curvature of the curved beam, where A, B, and C indicate the times corresponding to the three consecutive peak displacements.

This section first presents the solution procedure for the governing PDEs. Subsequently, the results obtained for two loading scenarios of the hyperelastic curved beam are presented using the average material parameters and compared with the 2D FEM solutions. In the first case, a prescribed vertical displacement was applied at the free end of the beam, according to the boundary conditions. The resulting deformation profile of the curved beam is illustrated in Figure 2. In the second case, cyclic displacement loading was applied to investigate the Mullins effect, and the corresponding deformation behavior is shown in Figure 3. For both loading scenarios, the stress distributions, including the non-dimensionalized Second Piola–Kirchhoff (${\bar{S}}_{ij}=S_{ij}/c_{1}$) and Cauchy stresses (${\bar{\sigma }}_{ij}=\sigma_{ij}/c_{1}$), are presented, and were evaluated and compared with the results obtained from finite element simulations. Subsequently, after presenting the results using the average material parameters, a probabilistic damage distribution was determined. For each material parameter, 100 values were generated following a normal distribution, and the corresponding probabilistic Mullins damage distribution was obtained through consecutive simulations. The mean values and the standard deviations of the material parameters are also presented in Table 1.

### 3.1. Solving Method

To yield a unique solution for the governing PDEs, appropriate boundary conditions must be prescribed. As illustrated schematically in Figure 1, the curved beam is subjected to a prescribed vertical displacement at its loading end while being rigidly clamped at the opposite end. Additionally, the inner and outer arcs are treated as traction-free surfaces. For complete clarity and exact mathematical replication, the detailed geometric locations and corresponding displacement constraints for all boundaries are systematically summarized in Table 2.

**Figure 4:**
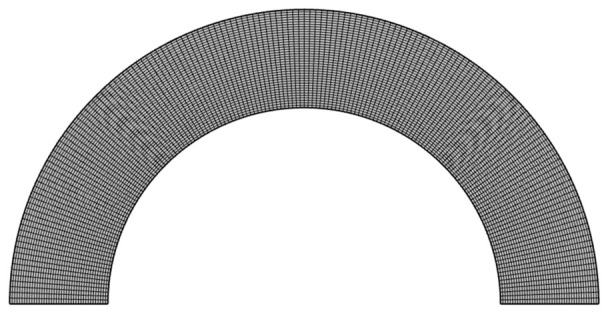
Swept mesh discretization adopted for both the PDE-based formulation and the 2D FEM.

The solution of this PDE was performed using the PDE solver in COMSOL Multiphysics (version 6.4). Specifically, the governing equations (Equations (25) and (26)) were implemented within the General Form PDE interface of COMSOL, and the simulation environment was finalized by prescribing the boundary conditions detailed in Table 2. For validation, the results were compared with a high-fidelity 2D FEM solution. In both simulations, a time-dependent study was carried out using the MUMPS direct solver, which was selected due to its exceptional robustness and efficiency in solving highly coupled, large-scale linear systems without numerical stability issues. Furthermore, the severe geometric and material nonlinearities induced by the hyperelastic Mullins effect were handled via the Newton–Raphson iterative scheme, ensuring quadratic convergence throughout the cyclic loading steps. Due to the inherent geometric regularity of the curved beam, a swept mesh was adopted for spatial discretization to maintain high element quality and reduce computational cost, as shown in Figure 4. To rigorously control the mesh dimensions and guarantee convergence, a Distribution feature was applied along both the radial and angular directions. The mesh convergence study was performed by increasing the number of elements in these two orthogonal directions until the critical stress and displacement fields independent of the mesh density were achieved. The PDE solver in COMSOL proved to be significantly faster than the 2D FEM approach, and this difference in computational time becomes especially noticeable in parametric sweep simulations used to obtain statistical distributions. COMSOL was thus employed to efficiently solve the equations.

### 3.2. Stress Distribution of Curved Beam

**Figure 5:**
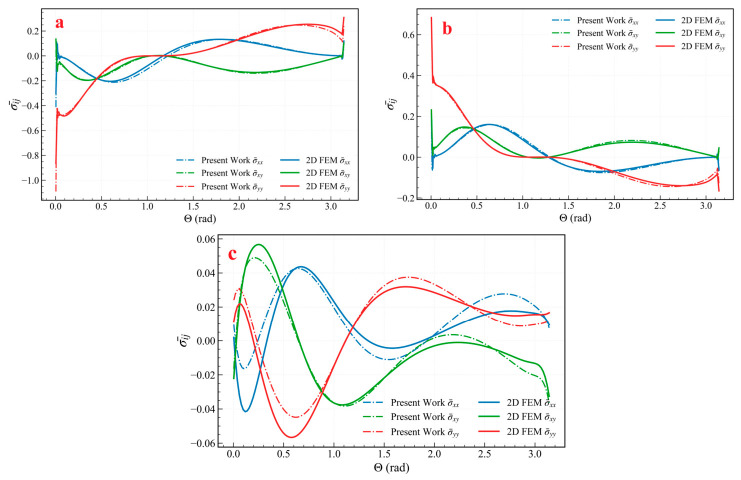
The non-dimensionalized Cauchy stress components along the beam thickness at (**a**) outside; (**b**) inside; and (**c**) middle arc.

After applying the prescribed displacement ($u_{y}=0.8R$), the non-dimensionalized Cauchy stress components in the Cartesian coordinate system were evaluated at three locations along the beam thickness (H): the outer arc, inner arc, and the mid surface of the curved beam. The corresponding stress distributions are illustrated in Figure 5.

**Figure 6:**
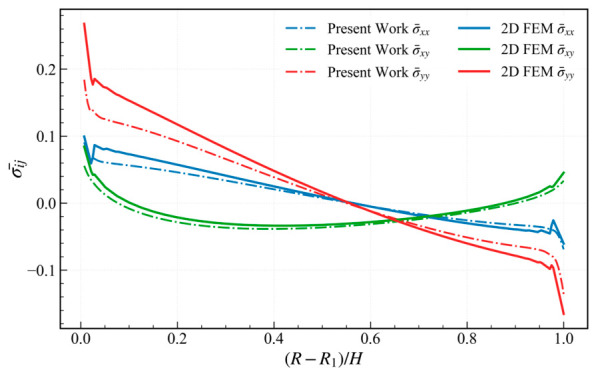
The non-dimensionalized Cauchy stress components at the end cross-section of the curved beam.

Furthermore, the non-dimensionalized stress field at the end cross-section of the curved beam is presented in Figure 6, where the variation in stress across the thickness and along the curvature can be clearly observed.

**Figure 7:**
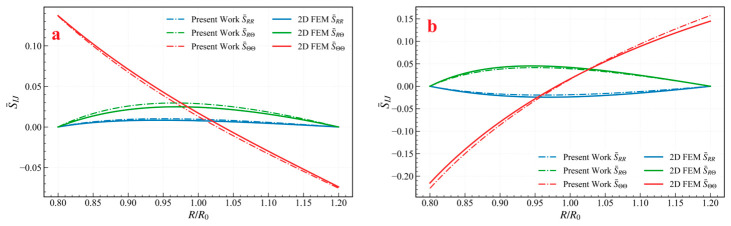
The non-dimensionalized Second Piola–Kirchhoff stress components at angular positions of (**a**) $90^{\circ }$ and (**b**) $45^{\circ }.$

**Figure 8:**
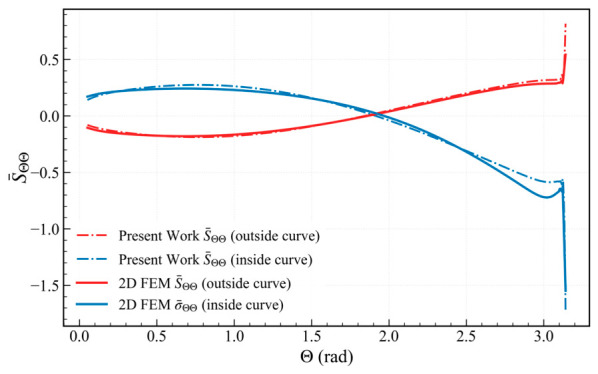
The non-dimensionalized hoopstress at both the inner and outer arcs of the curved beam.

The non-dimensionalized Second Piola–Kirchhoff stress components in the cylindrical coordinate system were evaluated at several cross sections of the curved beam, specifically at angular positions of $45^{\circ }$ and $90^{\circ }$. The corresponding stress distributions are presented in Figure 7. In addition, the hoop stress at both the inner and outer arcs of the beam was examined (Figure 8). As expected for the applied loading and boundary conditions, the radial and shear stress components vanish at these free surfaces, while the circumferential normal stress exhibits significant variation across the beam thickness.

According to the results presented, as expected, the curved beam unlike straight beams exhibits nonzero stress in the radial direction with a nonlinear distribution across the thickness. This specific radial stress, combined with the induced in-plane shear stress, can be highly detrimental to the structural integrity of the beam. Under large deformations, the coexistence of substantial radial tensile and shear stresses generates a complex triaxial stress state. In thick hyperelastic structures, such a stress state is known to trigger internal cavitation, micro-void coalescences, and localized tearing, ultimately leading to catastrophic interlaminar failure or delamination. Ignoring these non-zero radial and shear components as done in simplified pure-bending or thin-beam theories results in a dangerous underestimation of the localized failure risk, especially near the inner curvature. Moreover, the proposed solution maintains high accuracy and a low margin of error, provided that the vertical displacement does not significantly exceed the initial radius of curvature ($\frac{u_{y}}{R}\gg 1$).

**Figure 9:**
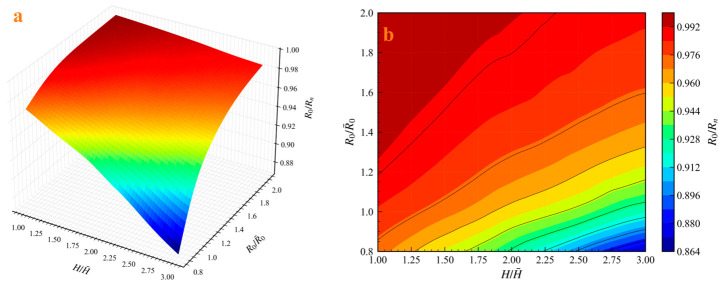
The coupled effects of normalized thickness ($H/\bar{H}$) and normalized initial radius ($R/{\bar{R}}_{0}$) on the shifting of the neutral axis ($R_{0}/R_{n}$) presented in 3D surface (**a**) and 2D contour (**b**) plots.

As previously mentioned, the neutral axis in curved beams deviates from the cross-sectional geometric centroid. To demonstrate the coupled effects of curvature and thickness on the shift of the neutral axis, the results are illustrated in Figure 9. This figure presents the ratio of the neutral axis curvature radius $\left(R_{0}/R_{n}\right)$ across various configurations, where the thickness and initial radius ($R_{0}$) are normalized by the reference thickness ($\overset{¯}{H}$) and reference radius ($\bar{R_{0}}$), respectively. According to the obtained results, a noticeable discrepancy of up to 14% is observed between the neutral axis and the geometric centroid in certain cases, highlighting the significant structural divergence of curved beams from straight beams. Consequently, this neutral-axis shift displaces the location of maximum stress and introduces significant design complexities, rendering conventional straight-beam approximations highly inadequate for thick curved parts.

### 3.3. Mullins Effect Under Cyclic Displacement

**Figure 10:**
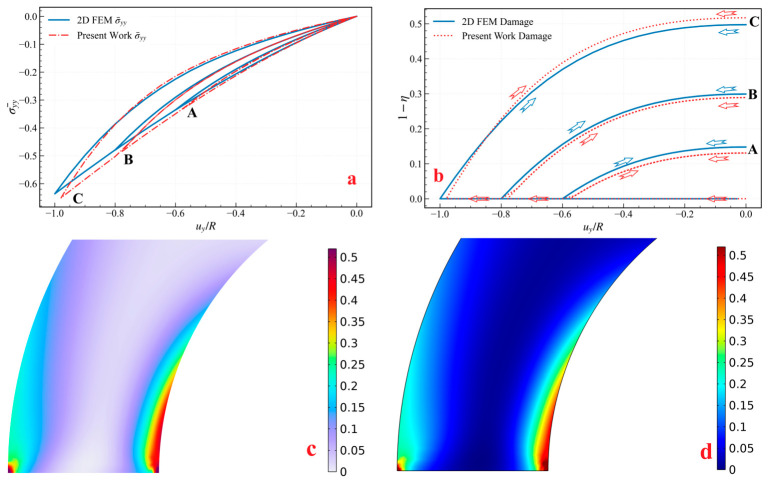
Comparison of stress, damage evolution, and contours: (**a**) Cauchy stress $\sigma_{yy}$ versus normalized displacement at the inner root of the curved beam. (**b**) Maximum damage at the root versus normalized displacement, where the arrows represent the direction of the cyclic loading and unloading path. (**c**) 2D FEM Damage contour for the entire geometry. (**d**) Damage contour obtained from the present work.

This section presents the Mullins damage response of the hyperelastic curved beam under cyclic loading. The evolution of the damage parameter during cyclic loading is presented in Figure 10, where the progressive reduction in stiffness with each loading cycle can be observed. The maximum stress at the beam root after three loading cycles is illustrated in Figure 10, clearly demonstrating the stress-softening effect induced by the Mullins mechanism. Furthermore, the damage contour is shown in Figure 10, highlighting the regions within the curved beam that experienced the highest level of degradation by the end of the loading cycles. As expected, the damage is concentrated near the inner curvature and the beam root, where the stress intensity is highest. This severe localization occurs because these regions bear the highest stress gradients under bending, making them the primary critical zones during cyclic operations. Under sustained cyclic loading, this localized stress-softening leads to a progressive reduction in the macroscopic stiffness of the entire structure. If not properly accounted for, this stiffness degradation accelerates structural softening and can drastically shorten the component’s operational life. Therefore, the proposed framework provides essential data for predicting these failure-prone zones, allowing designers to implement optimized safety factors and achieve safer geometric designs for curved hyperelastic components.

**Figure 11:**
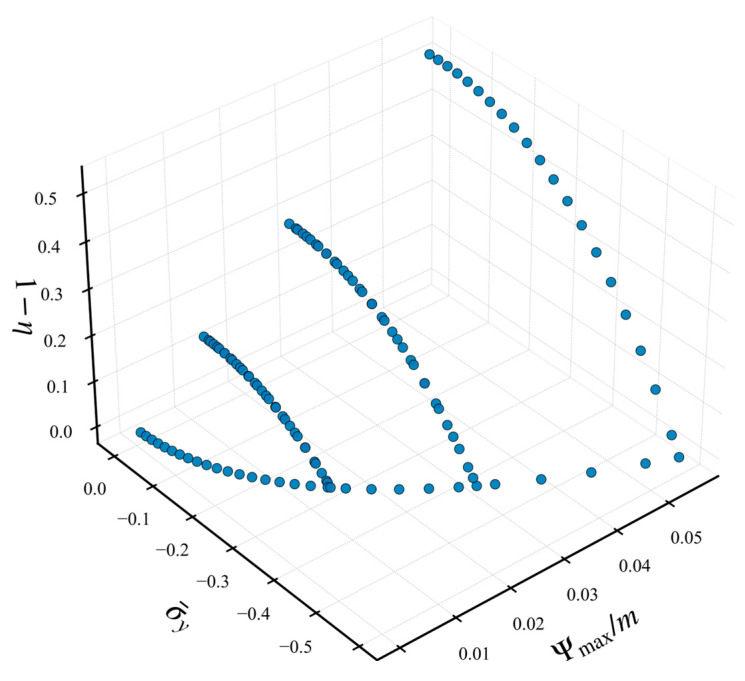
3D plots of the non-dimensionalized stress, and the maximum energy function.

Moreover, for better visualization of the results, 3D plots of the non-dimensionalized stress, the maximum energy function (Equation (19)), and the Mullins damage value are presented (Figure 11).

### 3.4. Probability of the Mullins Effect

**Figure 12:**
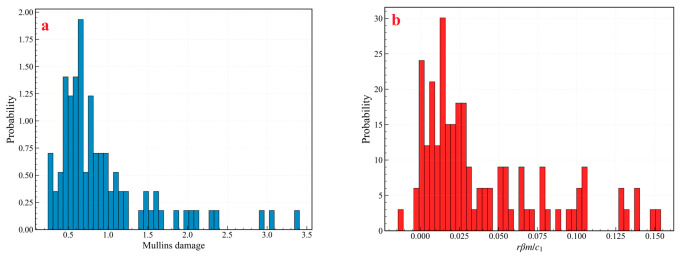
Histogram of Mullins damage (**a**) and damage parameter (**b**) probability.

A total of 100 random sets of material parameters, generated according to the normal distributions reported in Table 1, were simulated using a parametric sweep. The results are presented as histograms (Figure 12). The advantage of reduced simulation time using the theory presented here becomes evident: For simulating 100 separate runs, which would normally require high-performance servers or several hours, the results can be obtained in just a few minutes (19 min).

According to the results presented in the histograms, the damage ranges from 0.24 to 3.4, indicating the influence of material variability on the Mullins effect. Moreover, the damage follows a normal distribution with a mean of μ=0.93 and a standard deviation of σ=0.63. For completeness, the skewness and kurtosis were also evaluated, yielding a skewness of 1.93 and a kurtosis of 3.81.

This statistical dispersion underscores the high sensitivity of the structural degradation to the inherent variability of the material properties. Although the input parameters are governed by symmetrical distributions, the strong positive skewness (1.93) in the resulting Mullins damage implies a prominent right-skewed tail, indicating a non-negligible probability of encountering specimens that undergo unexpectedly higher damage under identical cyclic loading. Conversely, the high positive excess kurtosis (3.81) reflects a leptokurtic distribution with a sharper, more distinct peak around the mean (0.93), suggesting that while a significant portion of the damage values closely clusters around the average, the intense coupling between the non-linear material behavior and the curved beam geometry generates heavy tails that lead to an extended scattering range. From a structural reliability perspective, this broad operational range emphasizes that relying solely on deterministic, average-based predictions would fail to capture critical upper-bound risk limits.

These findings clearly indicate that although the majority of responses occur around the mean value, the inherent stochastic nature of material properties demands that engineers always incorporate a conservative, worst-case scenario perspective into the design and analysis. Consequently, the proposed probabilistic framework successfully quantifies these stochastic variations, which are essential for establishing conservative safety factors and high-fidelity service life predictions for polymeric curved structures.

## 4. Conclusions

This study presents a comprehensive analysis of hyperelastic curved beams under cyclic displacement, incorporating the Mullins stress-softening effect and probabilistic material variability. The key findings are as follows:

1. The Mullins effect induces progressive softening of the beam, with damage predominantly concentrated at the inner curvature and beam root.
2. The developed PDE-based model shows excellent agreement with 2D FEM results, validating the proposed approach.
3. Material variability significantly affects the Mullins damage distribution, as demonstrated through probabilistic simulations and statistical analysis.
4. The proposed framework enables fast and accurate evaluation of the mechanical response of hyperelastic curved beams, which can be utilized in the design of polymer-based structures with improved durability absorption.

Overall, the study provides an efficient computational methodology for predicting the cyclic behavior of hyperelastic curved beams, integrating both nonlinear hyperelasticity and statistical variability of material parameters, offering a valuable tool for engineering design and optimization.

## Figures and Tables

**Table 1:** Material properties and stochastic parameters of the rubber [50].

	$c_{1}$ (MPa)	r	m (MPa)	β
Mean	1	1.1	0.15	0.25
Standard deviations	0.15	0.5	0.1	0.1

**Table 2:** Boundary conditions of Curved Beam.

Beam Boundary	Geometric Location	Constraint
Loading End	$-1.2<\frac{R}{R_{0}}<-0.8\;and\;\Theta =180$	Prescribed: $u_{y}=\Delta$ Constrained: $u_{x}=0$
Clamped End	$-1.2<\frac{R}{R_{0}}<-0.8\;and\;\Theta =0$	Fully Fixed: $u_{x}=0,\;u_{y}=0$
Inside Arc	$\frac{R}{R_{0}}=-1.2\;\mathrm{and}\;0^{\circ }\leq \theta \leq 180^{\circ }$	Traction-Free: S⋅N=0
Outside Arc	$\frac{R}{R_{0}}=-0.8\;\mathrm{and}\;0^{\circ }\leq \theta \leq 180^{\circ }$	Traction-Free: S⋅N=0

## Data Availability

The original contributions presented in this study are included in the article. Further inquiries can be directed to the corresponding author.
